# Promoting outdoor recreation among older adults in Sweden – a theoretical and empirical foundation for the development of an intervention

**DOI:** 10.1186/s13690-021-00762-6

**Published:** 2021-12-27

**Authors:** Magnus Zingmark, Rosemarie Ankre, Sandra Wall-Reinius

**Affiliations:** 1 Municipality of Östersund, Health and Social Care Administration, 83182 Östersund, Sweden; 2 Department of Health Sciences, Lund University, Lund, Sweden; 3 Department of Epidemiology and Global Health, Umeå University, Umeå, Sweden; 4 Department of Economics, Geography, Law and Tourism, Mid Sweden University, Östersund, Sweden; 5 European Tourism Research Institute (ETOUR), Mid Sweden University, Östersund, Sweden

**Keywords:** Accessibility, Active ageing, Complex intervention, Disengagement, Health, Quality of life, Nature, Mobility, Life-space

## Abstract

**Background:**

Disengagement from outdoor recreation may diminish the positive benefits on health and well-being in old age. The purpose of this study is to present a contextual, theoretical, and empirical rationale for an intervention, aiming to promote continued engagement in outdoor recreation for older adults in a Swedish context.

**Methods:**

The paper includes a contextualization of outdoor recreation in Sweden, a presentation of evidence on health benefits related to engagement in outdoor recreation, together with theoretical frameworks that may guide future intervention designs. To add empirical knowledge, a mixed methods approach was applied, including an empirical data collection based on a quantitative survey (*n* = 266) and individual semi-structured interviews with older adults (*n* = 12). Survey data were presented with descriptive statistics. Associations between disengagement from previously performed activities and age and gender was analyzed with Chi^2^ tests. Transcripts and handwritten notes from the interviews were analyzed qualitatively to identify key themes, as well as patterns and disparities among respondents.

**Results:**

Outdoor recreation was rated as important/very important by 90% of respondents of the survey. The interviews highlighted that engagement in outdoor recreation aided respondents to keep fit but had also relevance in terms of identity, experiences, and daily routines. Outdoor recreation close to the place of residence was most common and walking was the most frequently reported activity. While 80% considered their health to be good/very good, disability and long-term diseases were common and during the previous year, more than half of all respondents had disengaged from activities previously performed. Reasons for disengagement were mainly related to health decline or that activities were too demanding but also due to social loss. The interviews indicated that continued engagement was important but challenging, and that disengagement could be considered as a loss or accepted due to changing circumstances.

**Conclusions:**

In the design of an intervention aiming to promote engagement in outdoor recreation for older adults, the following features are proposed to be considered: person-centeredness, promoting functioning, addressing self-ageism, providing environmental support, promoting subjective mobility needs and adaptation to find new ways to engage in outdoor recreation.

## Background

As the number of older adults increase, policies and actions on active ageing becomes an increasingly important societal issue [1]. From a societal perspective, active ageing refers to the processes of optimizing opportunities for health (physical, mental, and social), participation and security. This includes opportunities in relation to accessibility (e.g., transportation, housing, outdoor environments, services) and inclusion (e.g., anti-ageism, volunteerism) [1]. From an individual perspective, active ageing has been described as “*the striving for elements of well-being through activities relating to a person’s goals, functional capacities and opportunities*” [2]. Taken together, both societal and individual aspects are relevant to consider when initiating actions to promote active ageing. In this paper, we focus on one specific aspect of active aging: outdoor recreation. Continued engagement in valued activities is a marker of active and healthy ageing as described by older adults [3, 4]. There is also evidence that an active lifestyle positively affects disability and morbidity [5–7], well-being [8], and reduces mortality risk [9]. The benefits of engagement in outdoor recreation include for example reduced stress, improved cognitive ability and mental health, promotion of physical activity and social participation [10, 11]. However, with increasing age, older adults are less likely to take an active part in outdoor activities due to limitations in health, personal safety, physical capacity, and disability [12]. If such limitations lead to involuntary disengagement from outdoor recreation, the benefits on health and well-being may also be negatively affected. Thus, there is a need to explore how older adults’ engagement in outdoor activities evolves over time with focus on how involuntary disengagement can be addressed.

This paper is part of a larger project about accessible and inclusive outdoor environments in Sweden with the overall goal of increasing knowledge about accessibility, lifestyle and physical activity among older adults and people with physical disabilities. Of particular interest to the project is to identify how accessibility and social inclusion can be strengthened in the local community and thereby promote citizens’ health through equal access to outdoor environments. With this research, we want to better understand factors that enable and hinder that development. In this paper, we focus on the first development stages of an intervention and active ageing from an individual perspective. Based on the Medical Research Council’s framework for developing and evaluating complex interventions, we hypothesized that an intervention focused on promoting engagement in outdoor recreation for older adults can be considered complex. That is, such intervention is likely to include several components that may be tailored individually, and several outcomes may be affected by the intervention [13]. The purpose of this paper is to present a contextual, theoretical, and empirical rationale for an intervention, aiming to promote continued engagement in outdoor recreation for older adults in a Swedish context.

In the following, we (i) contextualize outdoor recreation in Sweden, (ii) present evidence on the health benefits related to engagement in outdoor recreation, (iii) present theoretical frameworks that may guide the design of an intervention to promote engagement in outdoor recreation for older adults. In addition, (iv) the methods and results from a mixed-method study (questionnaire survey and semi-structured interviews) is presented. We end the paper by discussing and concluding main results. Core features are proposed to be considered in intervention design to promote engagement in outdoor recreation for older adults.

### Outdoor recreation, health and well-being

The importance of public health, recreation and quality of life has been evident ever since outdoor recreation in Sweden developed in late nineteenth century; the transitional period of industrialization and urbanization. To be in nature and take part of outdoor activities, is a cultural cornerstone and component of the Swedish identity [14]. There is also a strong tradition of viewing contact with nature as a democratic right; nature should be accessible to everyone which also is implemented in present Swedish politics and government guidelines. Being in a natural environment may have beneficial effects on both physical as well as psychological health [15]. As Payne et al. (2005) state, local parks create space for physical activities; these nature areas should be considered as a part of a strategy for health promotion and disease prevention among older adults [16]. The concept outdoor recreation hence includes experiences of nature that has implications for health and well-being but is broad regarding both the type of activities engaged in as well as the types of nature environments [17]. In this article, we define nature as forests, mountains, and coastal areas but also urban green areas.

In Sweden, nature experiences and outdoor recreation play a significant role in many people’s life in terms of daily routines, identity, and tradition [18]. Outdoor activities are hence highly valued by many Swedes who engage in some form of outdoor recreation; more than half of all Swedes states they visit the nearest nature area at least every week [19]. In Sweden, the Council for Outdoor Recreation (2010:2008) defines outdoor recreation as “*to be outside in natural or cultural landscapes for well-being and encounters with nature without demands for competition*”. The most common outdoor activities are found in the relatively simple activities in terms of walks, to be in the forests and fields, outdoor swimming, picknicks, and cycling. The relevance of engagement in outdoor recreation can be understood in relation to health and well-being e.g., physical activity, increased mental health, reduced stress, and less exposure to pollution [20]. In addition to physical and mental health effects, spiritual experiences have also been highlighted, for example, the feeling of being connected to something greater, experience of freedom, or believing nature as life-giving [21]. People visit nature and engage in outdoor recreation for various reasons e.g., to experience nature environments and beautiful landscapes, but they also want to escape the everyday life and get peace and quiet. Others want to get exercise and improve their health and self-esteem, or spend time with family and friends [22].

Outdoor recreation as part of active ageing can be seen in relation to that 20% of Sweden’s total population is 65 years or older and the proportion of older people in the population will increase for the decades to come. Engagement in leisure, including outdoor activities, is positively related to self-rated health, well-being [23, 24] and survival [24, 25]. In a longitudinal study of Swedish older adults’ leisure activities and quality of life [26], older adults who increased their activity participation, e.g., walking more frequently, tended to feel that their life quality had increased, especially among those who were lonely or had less physical ability. Over time, participation in leisure activities among older adults has increased [27], indicating that leisure is an important part of older adults’ lives. According to a national survey conducted in 2018, Swedish older adults + 65 are much more often in nature in their everyday life, compared to those who are 16–44 years old. Common activities are walks for pleasure and exercise, gardening, and being outdoors and in forests for a nature experience. In addition to the health benefits described above, being in nature gives experiences such as tranquility and possibility of recovery [19]. I The recent generation of older adults is not only healthier than previous generations, but a larger proportion also has the time and money to engage in travel, tourism and outdoor activities [28, 29].

In the relationship between activity engagement, health, and well-being as described above, the experience of meaning is one important component [30–32]. Further, the experience of health is related to the balance between a person’s ability to act and to what extent he/she can fulfill subjectively meaningful goals [33]. The experience of meaning has been described in terms of doing, being, becoming and belonging [32, 34]. Beyond performance of activities (*doing*), the experiences of engagement in activities – such as outdoor recreation - provides a source for self-reflection that contributes in shaping a sense of identity (*being*) and relationships to others (*belonging*) as manifested in social roles [32, 34–36]. *Becoming* has been described as “an ever-incomplete process” [34], highlighting that throughout life there is an ongoing process, requiring that the person deals with new opportunities, challenges and demands to achieve well-being derived from being engaged in activity. In all, the opportunity to engage satisfactorily in a mix of activities that are subjectively meaningful, provides a potential link between occupation, health, and well-being [31, 33, 34, 36, 37].

### Theories and models related to engagement in activity in old age

A set of established theories from social gerontology were chosen to provide an understanding of how engagement in activity may transform and be promoted during old age. The continuity theory and disengagement theory provide an understanding of engagement in activity during the ageing process. In the *continuity theory,* continuity is seen as a main strategy for coping and adapting when adverse events occur (e.g., disability) [38]. According to the continuity theory, older adults who experience adverse events tend to maintain values, lifestyles and relationships based on the preferences developed throughout their lives [38, 39]. In contrast, *disengagement theory* suggests that older adults tend to adapt their engagement towards fewer activities and social roles in a narrower context compared to their earlier life. Disengagement theory specifically relates to a transformed pattern of interaction rather than the quality and amount of activity [40]. While continuity and disengagement provide with two perspectives to understand engagement during old age [38], there is also a need to consider how engagement can be modified. This can be explained based on the following three models.

The *Selection, Optimization, Compensation* (SOC) model [41, 42] encompasses the identification of vital goals (*Selection)*; how skills and resources can be maintained, improved or acquired in order to achieve those vital goals (*Optimization*); and how different strategies may compensate for the lack of skill or resources, in order to maintain a desired level of functioning (*Compensation*). Over time, and especially in the event of health decline, optimization becomes increasingly challenging [41]. Therefore, the selection and compensation become increasingly importantas means to maintain functioning and engagement in valued activities, but also to direct efforts to improve engagement in prioritized domains. The *Comprehensive Preventive Corrective Proactive* (PCP) model depicts how older adults cope with or adapt to age-related challenge [43]. The specific feature of the PCP model is its focus on proactive behavioral adaptations that stems from the idea that age-related challenges (morbidity, disability and social loss) are common. Thus, actions can be taken in advance in order to reduce risks and be better prepared for such challenges. Examples of proactive behaviors are engagement in healthy lifestyles (e.g., eating a healthy diet, exercising) and planning for the future (e.g., relocating to an accessible living environment). In addition to proactive actions, the PCP also addresses current challenges such as finding new roles after social loss, or modifying the environment due to disability [43]. In relation to continued engagement versus disengagement in/from outdoor recreation, the SOC and the PCP-models describe individual strategies as well as contextual factors. These have the potential to optimize a person’s engagement and promote active ageing. A third model, the Person-Environment-Occupation (PEO) model depicts the dynamic interplay between the person (e.g., capabilities, personality), the environment (e.g., physical, social) and specific occupations (i.e., the valued activities a person engages in) [44]. In line with the PEO model, a mismatch between a persons capabilities, the specific activities that he/she wishes to engage in, and a challenging outdoor environment (e.g., uneven, hilly terrain) may limit the opportunities for engagement.

In all, given that outdoor recreation is important for many older adults, activities in nature have the potential to impact health and well-being and, ultimately, promote active ageing. However, there is a need of better understanding of how the patterns of engagement in outdoor activities develop over time and to identify different factors that have an impact on a continued engagement. In the sections below, we present the methods and results from a mixed-methods study conducted in a Swedish municipality.

## Methods

To add empirical knowledge from a local perspective to the rationale for the development of an intervention, a mixed methods approach was applied, including an empirical data collection based on a quantitative survey and individual semi-structured interviews with older adults.

### Setting

The empirical data collection took place in the municipality of Östersund, Sweden. The area of the municipality is 2502 km^2^ and at the time for this study (2020), the municipality had about 64,000 citizens of which 21% was 65 years or older. The city of Östersund is built around Sweden’s fifth largest lake, Storsjön, where boreal forests and small scale-scale agriculture characterize the rural areas surrounding the city. There are several nature reserves within the municipality, and in its vicinity. About an hour’s drive from the city, the Scandinavian mountain range is to be found. The climate is characterized by a winter period including snow and ice from November to mid-April, and a summer period from late May to early September. Since 2019, the municipality is a member of the World Health Organizations global network *Age-friendly Cities and Communities*. In relation to this membership, the municipality has initiated a range of activities to introduce more health-promoting and preventive measures for older adults and people with disability.

### Data collection, participants, and analysis

#### Quantitative data collection and analysis

During the winter and spring of 2020, a quantitative survey was distributed in paper form (handed out personally by the researchers), to randomly selected participants at a local senior exhibition accessible free of charge for the public (25–26 January 2020) and at two different meetings organized by pensioners’ associations (25 February and 10 March 2020). These events occurred before COVID-19 restrictions were initiated in Sweden by mid-March. At these events there was a variety of people including men and women from young old to more advanced ages. The inclusion criterion was age 65 years or older. The survey consisted of 16 questions, both closed and open questions. The survey took about 15–20 min to answer. The questions concerned experiences of being in nature, engagement: type of outdoor activities engaged in, when (time of year, weekdays, weekends, holidays), frequency (how often), and if the respondent mainly spent time in nature in solitude or together with others, health, physical activity, and as perceived accessibility**.** The survey also included a question about how nature areas in the municipality could become more accessible. Another question concerned if the respondent had any disability (e.g., impaired mobility, vision, or hearing) or a long-term disease (e.g., diabetes, cardiac or respiratory disease), that could have an impact on the opportunities to participate in outdoor recreation. In addition, demographic data was collected (year of birth and gender). The survey was anonymous in terms that no name, personal identification number or contact information was collected.

The questionnaires were coded and entered in IBM SPSS (*Statistical Package for the Social Sciences*). There were missing data on single questions and thus for each question, the numbers of respondents, as well as proportions, are reported below. The data were analyzed and presented by using descriptive statistics. To explore if involuntary disengagement was associated with age and/or gender, Chi^2^ tests were used to analyze the impact on respondents´ ability to engage in previously performed outdoor activities to the extent they wished during the previous 12 months.

#### Qualitative data collection

During the fall of 2020, we conducted individual, semi-structured interviews. The development of the interview guide was informed by preliminary findings from the survey. The interview guide included questions about the respondents’ perceptions of outdoor recreation and nature, their health, reflections regarding aging, how they experienced that their participation in outdoor recreation had changed over time, as well as their experiences and needs of accessibility, safety, and information. Follow-up questions were used, often leading to an unstructured dialogue where issues and anecdotes emerged. This procedure gives the respondents control over the interview [45], and lead to rich descriptions, statements, and examples of how the respondents think about outdoor activities, health, and well-being.

The three authors alternated as interviewers, where two always participated together to ask questions and take notes. Due to COVID-19 and pandemic-related restrictions, physical meetings with potentially vulnerable older people were inappropriate. Therefore the interviews took place by telephone which nevertheless is a successful way of getting knowledge without reducing data quality [46]. To ensure the quality of the interview, the interviewers sat together in a room without disturbing noises. Before the interview commenced, the interviewers made sure the interviewees could hear what was said. The interviewees were informed that they at any time could discontinue the interview. The interviews lasted approximately one hour, where every interview was recorded and transcribed. The transcripts and handwritten notes were carefully read, where we identified key themes and ideas, as well as patterns and disparities [47] among the interviewed older adults.

The interviewees were identified by a snowball method, i.e., two different local pensioners’ associations and organizers for municipal meeting places for older adults were asked to identify and ask older adults about interest in the study, and to inform the researchers of potential interviewees. Inclusion criteria were age 65 years or older and that the person was capable of outdoor activities on their own. Twelve older adults (four men and eight women aged 75–89) consented to participate, see Table 1. Among these, nine participants had lived in the local area for most of their lives, while three individuals had moved to the area later in life. Half of the participants had access to a car, while the other half had not due to health issues or age. These participants used public transport or special transportation services for people with disability instead. The participants varied regarding their level of functioning; four of the interviewed had a walker as an aid. Some had quit biking because of problems with balance, while others still went cross-country skiing or walking in challenging terrain e.g., in the mountains.

**Table 1:** Overview of participants in the qualitative interview

Interviewed older adults*	Sex	Age	Marital status	Access to car	Need of support/walker
Inga	Female	80	Widow	No	No
Eva	Female	89	Widow	Yes	No
Siv	Female	81	Widow	No	No
Anita	Female	75	Widow	Yes	No
Bengt	Male	76	Married	Yes	No
Karin	Female	81	Widow	No	Yes
Hans	Male	82	Lives alone	No	Yes
Lars	Male	75	Married	Yes	No
Ingrid	Female	86	Lives alone	No	Yes
Berit	Female	86	Widow	No	Yes
Karl	Male	80	Married	Yes	No
Birgitta	Female	75	Married	Yes	No

*Pseudonyms used

## Results

In all, 266 respondents answered the survey; 62.4% were women and the mean age was 74,7 years. The results from the survey and the interviews have been analyzed by patterns and themes. In the result section, the data sets are combined and presented by identified themes.

### Importance of outdoor recreation for health, well-being, and routines

The results from the survey show that 72.9% reported some form of disability or long-term disease that had an impact on opportunities to participate in outdoor recreation. When asked about perceived health status, however, 59.7% considered their health to be good and 19.8% as very good. Regarding the ability of physical mobility outdoors, 50.8% considered their ability to be good and 32.8% as very good. Outdoor recreation was rated as very important by 132 respondents (51.2%) and important by 99 respondents (38.4%); the remaining 27 respondents (10.5%) rated outdoor recreation as important to some extent. In the interviews, the importance of outdoor recreation was confirmed both in terms of the potential for promoting and maintaining health as well as in terms of everyday well-being. Lars, 75 years, described the value of outdoor recreation as:

"*It is refreshing, you have to keep your body and circulation going. The body feels good and the head feels good, from observing something natural. You follow the seasons, and you are part of the world and the biological cycle when you walk in nature*."

The interviewees expressed concerns to maintain their physical and mental health and an awareness of how outdoor recreation could contribute to that. Eva, 89 years, stated that:

“*The important thing for me is to be able to move my legs, so you keep the strength you have. I do not want to get lazy and sit at home and feel sorry for myself. You feel more free in some way. It is the greatest experience and joy that you can be outdoors, because you still can walk*.”

The results from the survey showed that around 90% of the participants often (*n* = 86, 34.8%) or very often (*n* = 134, 54.3%) spent time in nature within the municipality. However, 27 (10.9%) respondents reported that they never or very seldom (once per month) spent time in nature**.** In addition to being an important means to maintain health, several of the interviewees emphasized that being outdoors in nature was part of their identity and lifestyle, as well as part of their routines and habits. As Inga, 80 years expressed:

“*Nature is everything. I do not know how I would survive if I could not get outside. I dread if I get demented and locked up. It* [nature] *is a big part of my identity, I have always been outdoors. It is important*.”

Peace and tranquility were described as valued experiences and several respondents also mentioned being part of nature, and the importance of experiencing the change of seasons. Bengt, 76 years old, expressed the importance of being in nature in this way:

“*You learn to observe animals and plants, and you learn to appreciate nature. That is one reason. I watch a lot of plants and that nature changes color. Experiencing and paying attention to this, is a big event for me nowadays*.”

Berit, 86 years, continued:

“*It* [nature] *is very important; otherwise, you would not be able to survive. It would be very sad otherwise, if you were tied to the home and its four walls. It feels like you have to get outside when the weather is nice, in order to get fresh air*. "

### Engagement in outdoor recreation and changes over time

The activities that respondents reported that they engaged in were walking *n* = 200 (74.6%), cross-country skiing *n* = 67 (25.0%), picking berries *n* = 48 (17.9%), cycling, *n* = 44 (16.4%), and picking mushrooms *n* = 28 (10.4%). In addition, a range of other activities were mentioned by 14 respondents (5.2%) e.g., swimming, gardening, birdwatching, fishing, taking photos, and running. The interviewees confirmed that walking, especially in surroundings nearby the place of residence, was the most common outdoor activity. While some of the interviewees stated that this type of activity was at the top of their ability, others gave examples of long walks (+ 5 km) in the surroundings, frequent skiing in mountainous regions and bicycle tours. In line with the findings from the survey, the interviewees had experienced changes in how they engaged in outdoor recreation. Despite such changes, frequent engagement clearly was important as stated by Karin, 81 years:

"*I usually take a walk every day, but because I have pain in one leg, I have a bit of a hard time walking. I have a walker that I use. I walk about two kilometers or so*."

Siv, 81 years, also emphasized the importance of routine and the specific activity engaged in:

“*I am taking walks on the forest trails almost daily. I walk along the beach. … I walk about an hour a day. Earlier this year I bicycled. I think it is more exercise to walk than to bicycle. I namely have an electric bike*.”

More than half of the respondents in the survey (56.5%) answered that they had not been able to engage in previously performed outdoor activities to the extent they wished during the previous 12 months, Table 2. There were no differences between men or women in the proportion reporting disengagement (*p* = 0,677). When age was dichotomized in relation to the median (75 years), there was a significant difference indicating that a larger proportion of those 75 years or older (66.1 vs. 46.8%, *p* = 0,004) had not participated in one or more activities to the extent they wanted over the previous 12 months, compared to those younger than 75 years.

**Table 2:** Previously performed outdoor activities that respondents had not participated in during the last 12 months

Activity	***n*** (%)
Cross-country skiing	50 (18.7)
Cycling	19 (7.1%)
Downhill skiing	13 (4.9%)
Running	9 (3.4%)
Walking	8 (3.0%)
Hunting/fishing	7 (2.6%)
Picking berries	6 (2.2%)
Swimming/bathing	6 (2.2%)
Ice skating	5 (1.9%)
Picking mushrooms	4 (1.5%)
Trekking	2 (0.7%)

The activities presented in Table 2 indicate that disengagement from outdoor activities mainly concern activities that may be strenuous (e.g., cross-country skiing, running) or may be experienced as involving some risk (e.g., cycling, downhill skiing). As stated by Anita, a 75-year-old woman, health issues may cause involuntary disengagement as feelings of loss:

“*It is walking that I do. Nevertheless, you have ailments like everyone else. You have to move. Being outside affects me positively, when you are not - you get depressed, that is it. This year is the first summer I have not used the bike, it is sad, but at the same time, I have not felt like cycling. So, it has been okay anyway*.”

Even though disengagement from previously valued activities was expressed in terms of loss, there was acceptance of the changes that the interviewees had experienced. Hans, 82 years old, explained his feelings like this:

“*It was a long time since I went to Åre and Storlien* [the mountain areas]*, or since I went fishing. There is, of course, a yearning, but you have to accept the situation as it is and learn from the problems that arise. The interest in the things one would like to do, but cannot, diminishes. The physical capacity affects me the most, the mental capacity is no problem. I cannot sit here in the city and wish myself away, it does no good*.”

In the responses there were some, but few, indications of bitterness. One of the women, Ingrid 86 years old, described the following:

“*Nature has been very important to me, and I am happy for what I have received. Now I cannot be out in nature in the same way, due to severe visual impairment and a stroke which gave me mobility impairment. … Vision is a big obstacle that I have been needed to adapt to, and I try to enjoy other things that exist. Health rules over me now; before, I had the power. It* [health] *affects my approach to nature. You have to accept that there will be ailments.”*

### Factors that hinder or support outdoor recreation

Based on the survey, the most common reasons for not being able to participate in outdoor activities to the extent the respondents wanted, were changes in health or that the activity was too physically demanding, see Table 3.

**Table 3:** Reasons for not participating in outdoor activities

Reason	***n*** (%)
Changes in personal health	70 (26.1)
Too physically demanding	37 (13.8)
Too expensive	11 (4.1)
Insecurity	11 (4.1)
Lack of company to carry out the activity with	8 (3.0)
Weather/climate	8 (3.0)
Changes in partner’s health	6 (2.2)
Lack of access to suitable places/areas	6 (2.2)
Age	6 (2.2)
Lack of motivation/change in interests	5 (1.9)
Lack of transportation	4 (1.5)
Lack of equipment	3 (1.1)
Lack of information	3 (1.1)

The interviews confirmed that changes in health or activities being too physically demanding were reasons why some interviewees no longer were able, or could find the motivation, to participate in outdoor recreation the same way as before, as phrased by Lars:

“*Before, when you were younger, you had a stronger craving to experience things; now that I'm older it's not as exciting as I used to think. Then it is for sure, wobbling around on a boat when you are approaching 80 years old is not so good. It's a balance between risk and joy*."

Whereas some respondents described the feeling of unexploited nature as central for a positive experience of outdoor recreation, others clearly avoided uneven terrain such as trails with a lot of roots or hills. Instead, they emphasized the importance of paved roads and broad walking paths. Inga, 80 years, described different physical challenges in the landscape as stimulating that could help maintain physical function:

“Around Ändsjön* there are a lot of footbridges. It is obvious that there are roots in some places, but I think it is good training for the balance. It is varied, you take different length of the steps, you have to lift your legs more and less. It is good training.”

In contrast, Anita, 75 years, explained:

“*When I go into nature, I can walk in the woods, but you get wobbly over the years, so I stick to flat roads and paved paths. I do not walk in wild nature that much anymore; it is hard and difficult to walk. It is perhaps above all the risks of walking like this in nature, but also because it requires more of you.“*

Eva, 89 years, described how the terrain around Ändsjön was challenging but also had some thoughts on how accessibility could be improved:

“*Walking around Ändsjön is a balance exercise, there are many footbridges there. You have to be careful all the time; otherwise, it would have been a good path. If I was to suggest something, it is that as an older person, there are no places to sit or rest along the path*.”

**Fig. 1:**
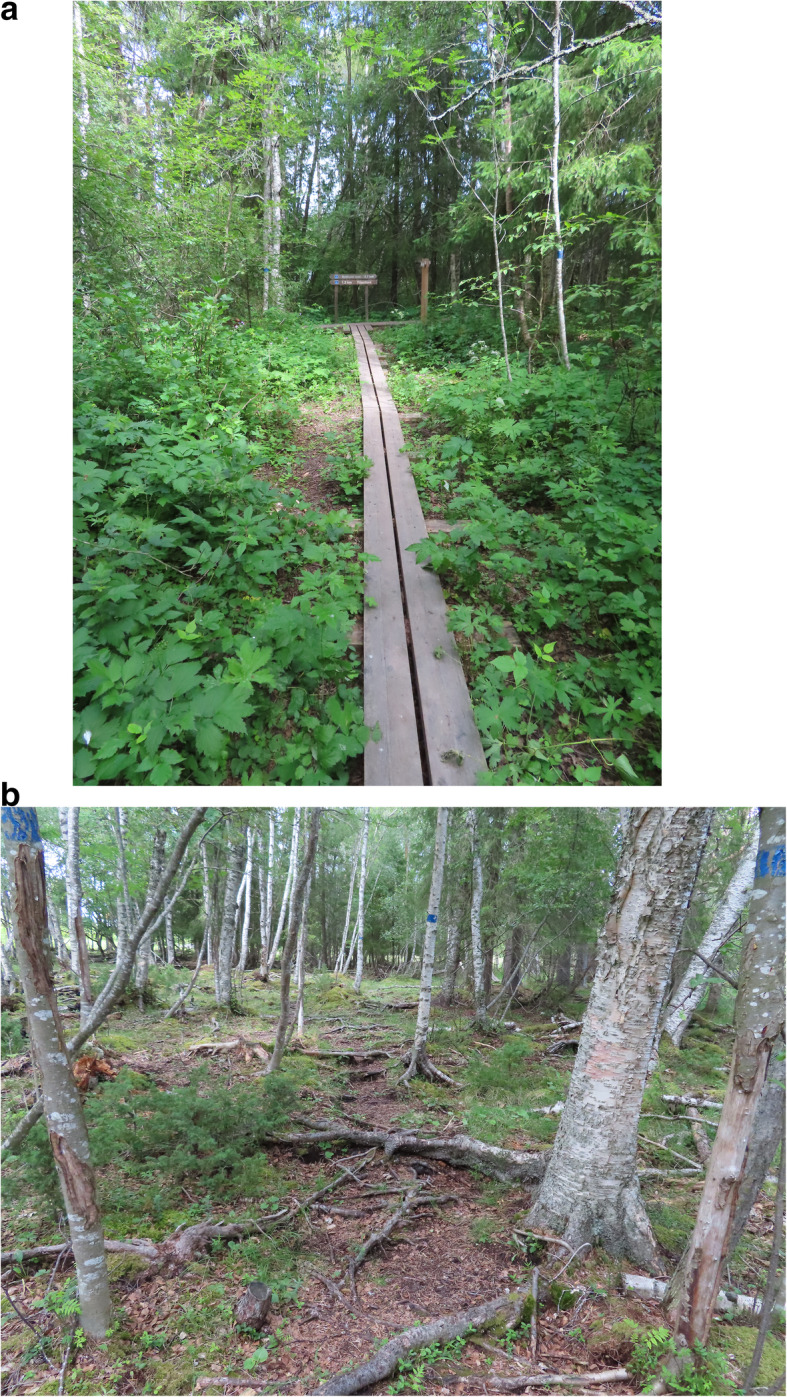
Ändsjön nature reserve and hiking trail summer 2020. Permission granted by copyright holder Rosemarie Ankre

*(insert as footnote) Ändsjön is a nature reserve of 1 km^2^ managed by the county administrative board and consists of a lake with a surrounding area situated about 5 km from the city center. The area is easily accessible by public transportation and car since there are several parking places. The 3 km hiking trail stretches around the lake and consists to some parts of footbridges, but several parts are quite challenging as described by the interviewees, see Fig. 1.

Even if some interviewees pointed out their need of a varied landscape with untouched nature, they also showed an awareness of other older people’s need for an adapted nature. Lars, who still practiced down-hill skiing and picked berries in the woods, had no problems with inaccessible nature. Nevertheless, he concluded:

*“All nature is not wild, much is a bit civilized so some nature could be adapted. … I think the accessibility to nature needs to be improved for those who do not live like me, to keep people healthier as they get older. There is a lot to do, but it also costs money. For example, to create more pathways and openings down to the lake.”*

Weather and climate were mentioned by a few respondents in the survey as reasons for not participating in outdoor recreation. In the interviews, these issues were highlighted from different perspectives. On one hand, “nice” weather did not seem to matter whether the respondents went outside or not, since they considered outdoor recreation was so important for their routines and well-being, so they got outside no matter what. On the other hand, seasonal change could be challenging, especially during the winter season due to ice and snow, which can make it difficult to walk. Siv explained that winter is challenging for her opportunities to walk outdoors:

"*You have to walk so damn carefully. The slipperiness in winter is disgusting. Even if you do not go that far, you get very tired because you are so tensed. Still, they* [the municipality] *are very good at sanding* [the roads*] around here*".

Few respondents answered that lack transportation was a factor that restricted engagement in outdoor recreation. The main mode of transportation to access nature environments was by walking (*n* = 136, 64.8%), by car (*n* = 60, 28.6%), by bike (*n* = 9, 4.3%), and by bus (*n* = 5, 2.4%).

Facilities such as adapted rest areas, and especially benches, were emphasized as important in order to get outside and hence being able to rest regularly. Interestingly, the interviews revealed that the benches were removed by the municipality in November and were not being put back until May, which increased their immobility during winter.

### Social aspects of outdoor recreation

One aspect linked to the older adults’ outdoor recreation was whether activities were performed in solitude or together with others. Based on the survey, the respondents usually spent time in nature alone (*n* = 102, 47.7%), together with a partner (*n* = 71, 33.2%), with friends (*n* = 31, 14.5%) or with children or grandchildren (*n* = 9, 4.2%). One respondent reported participation in organized outdoor activities e.g., by a senior organization.

The interviewees expressed that company was viewed as nice and fun, for example, a walk with a friend or with the local pensioners’ association. Given that several interviewees were recruited from such associations, there were several examples on how that social context provided a supportive context both in terms of arranged activities (such as walks or excursions), but also in terms of an inclusive atmosphere in which the level of challenge in the activity, e.g., pace and terrain, was adapted to fit all participants. A leader of a local pensioners’ association, Karl 80 years old, described the importance of planning activities to be suitable for participants with varying capabilities. He usually investigated the area before any organized hikes, which usually included 15–20 participants:

“*We do not want to go to the same places all the time, but to different places where it is convenient for us to go. It is quite easy to find such places.* [Critical factors] *are low level differences, however, we do not have participants with walkers but those who can walk by themselves. It is important that we walk on a forest path that does not become too narrow. It is very popular to use walking poles to keep balance and as an exercise tool, so then it is good if the paths are not too narrow*.”

Outdoor activities in solitude were also viewed as positive in order to get exercise, or to be able to choose a higher pace or more challenging terrain. Solitude also gave an opportunity to be alone with one’s thoughts. Inga clarified her satisfaction with her own company:

“*I am mostly myself. I kind of have no need of company to get outside. I am a widow so I live by myself. I think it is quite peaceful to be outdoors alone. I have no problem with that. In the fall, I am picking a lot of berries*.”

Among the interviewees who were widows, it was evident that not only health decline or challenging activities could affect the choice of outdoor activities, but also the loss of their husbands. The social change had an impact on their outdoor activities e.g., in terms that they had sold their second home. Eva described her situation after her husband had died:

“*We had his parents’ home … We were there a lot before. All that disappeared. It was a big change. He was ill a couple of years, so you got used to it a little before he died*.”

Also, regarding the inherent importance of nature, it became less important for Karin when her husband died:

"*Since I was left alone, it has not been the same. It* [nature] *had a great significance for me before … we were outdoor people both me and my husband*.”

### Places for outdoor recreation

In the survey, the respondents were asked to consider different factors that were important to them when choosing to visit a nature area in Östersund municipality. For two thirds of the respondents, closeness was considered important for their choice of nature areas in the municipality; a range of other factors is listed in Table 4.

**Table 4:** Factors considered important when choosing a nature area to visit in Östersund municipality

Factor	***n*** (%)
Close to one’s home	174 (64.9)
Marked paths and trails	108 (40.3)
Peace and tranquility	98 (38.6)
Familiar environments	87 (32.5)
Adapted rest areas, tables and benches	84 (31.3)
Safety	39 (14.6)
Mobile phone coverage	37 (13.8)
Opportunities for transportation	31 (11.6)
Information signs	31 (11.6)
Printed information	20 (7.5)
Non-adapted environment and pristine nature	19 (7.1)
Digital information	13 (4.9)
Other visitors	12 (4.5)

* Multiple choices were possible

To have nature areas nearby one’s home was very important according to the interviewees. As such, the place of residence is crucial for the opportunities to engage in outdoor activities. Two interviewees had actively undertaken moves from areas with an inaccessible outdoor environment with hills, to a flat area a few kilometers from the city center. This active choice resulted in improved opportunities for the two women to take daily walks. Ingrid concluded:

“*The changes and adjustments that I had to make - apart from a walker -, were to change accommodation to get closer to everything. ... When I got my stroke, I had to realize the end of my wonderful life in nature* [the countryside] *because it was not a place for me anymore - I had to give it up. I chose this area in Östersund city because I knew it was close to nature and I knew the area since I have lived here before*.”

There was no major interest in exploring new nature areas out of curiosity or adventure among the interviewed. Instead, familiar areas were considered more convenient and accessible. Consequently, the importance of information (printed or digital) was considered important by only a small proportion of the respondents. However, as a contrast, a few of the interviewees highlighted the digital nationwide orienteering challenge *HittaUt* [48] in which checkpoints, with varying degree of difficulty and terrain, are to be found within the local area with the help of a map (paper or digital). Birgitta, 75 years, had looked for checkpoints and emphasized how this helped her to get outdoors and to find new places. Karl had participated in the challenge for 3 years and found many checkpoints in different terrain. He believed it as suitable even for those with some difficulties to walk:

“*If you look at the areas in Björkbackaparken* [a city park], *you can get around with a walker and get the most checkpoints. There are different degrees of difficulty on the map*.”

## Discussion

Given the benefits on health and well-being related to engagement in outdoor recreation, and the high proportion of respondents reporting that they had not been able to engage in previously performed outdoor activities to the extent they wanted, there is clearly a need to explore how a continued engagement can be promoted. In the following, we discuss our findings and synthesize our results to propose a set of core features to be considered in the design of an intervention aiming to promote future engagement in outdoor recreation for older adults.

### Person-centeredness

The examples provided by our interviewees underline the importance of individual choice of outdoor activities as well as the specific contexts in which activities are performed. As indicated by the survey results, respondents reported a range of activities that they engaged in and a range of activities that they had disengaged from. Thus, an intervention aiming to promote continued engagement need to acknowledge and address the specific preferences of the person. Person-centeredness refers to an approach which involve active participation [36]. In line with the SOC model [41, 42], it is critical to identify the specific outdoor activities and contexts that an older person wishes to engage in. To direct the focus towards how to promote engagement in activities that are perceived as meaningful, provides a source for motivation. It may be used as a basis for goal setting. A high level of persistency in pursuing one’s goals and flexibility in order to adjust to changing circumstances, is beneficial for continued engagement in outdoor activities [49]. A clear structure for how to identify individual prioritized activities and context is necessary in order to apply person-centeredness for an intervention. There are several measures to achieve this, such as the Canadian Occupational Performance Measure (COPM). It provides an established approach for how to conduct an interview where prioritized activities are identified [50]. Furthermore, the AGNES study provides another example on how to use personalized goal setting to guide interventions aiming at promoting active ageing [51].

### Promoting functioning

In our study, the main reasons for disengagement from outdoor activities are health issues and/or that the activity is experienced as too demanding. While our data do not provide information on specific diseases or disabilities, it is reasonable that some health conditions can have a more severe impact on the capabilities to engage in outdoor recreation e.g., stroke or visual impairment as stated by one of the interviewees. Disengagement may arise suddenly due to severe injury or disease, or gradually e.g., due to reduced strength, poor balance, or fear of falling [52]. This may lead to a gradual reduction of engagement in outdoor activities, followed by a subsequent adapted self-image and expectations. Regardless the reasons for involuntary disengagement, an intervention to promote continued engagement in outdoor recreation needs to address factors such as physical functioning, self-efficacy, motivation as well as support for adaptation if previously forms of outdoor recreation are too challenging. Based on the SOC model [41, 42] and the PCP model [43], optimizing physical functioning is clearly relevant for older adults’ capacity and opportunities to engage in personally valued outdoor activities. While walking was the most prevalent outdoor activity, improved physical functioning could promote opportunities to maintain the ability to walk in more challenging terrain e.g., around Ändsjön, shown in Fig. 1, or to the ability to pick berries in the forest.

Few interventions have specifically targeted outdoor recreation, despite existing evidence which indicate that older adults’ health outcomes can be positively affected. Some interventions can be highlighted as potentially effective to promote outdoor activities. By a scoping review on preventive and health promoting interventions for older people in a Nordic context, a range of promising interventions (e.g., health promoting senior meetings, falls prevention, and physical activity counseling) can be identified to improve physical functioning [53]. For example senior group meetings have resulted in a number of positive effects, including walking ability and reduced fear of falling [54]. Personal advice on physical activity have also proved to have positive effects on advanced walking ability and maintained physical activity level [55]. Furthermore, while falls and fear of falling is a problem among the older population and a limiting factor in terms of outdoor activities, there is strong evidence that specifically designed strength and balance training, so-called OTAGO training, has positive effects on reducing falls and fall injuries [56]. In addition to optimizing an older adult’s functioning, compensatory strategies may also be considered as exemplified by Karin who used a walking stick to deal with pain while walking. Other technical aids that could be valuable in promoting engagement in outdoor activities could be a walker or a GPS-based safety alarm.

### Addressing self-ageism

One aspect related to functioning and what a person considers possible to do is his/her self-perception. Our results indicate that a large proportion of the participants were in challenging life phases. Hence, the patterns of outdoor recreation were in transitional phases as well. Diseases and disability were reported by a large proportion or respondents in the survey; half of all respondents stated that they had concluded their engagement in more challenging activities during the last year. Despite this, about 80% of the respondents rated their health, and ability to move around in outdoor areas, as good or very good. These somewhat conflicting results may be an indicator of gradually adapted preferences in the face of health decline and ageing [57]. While the disengagement theory describes the gradual withdrawal from activities and context during old age [40, 58], we suggest that it is important to acknowledge if these changes are involuntary or not, and if disengagement is experienced as a loss, as for Anita who had finished cycling. Changes in health or activities being too physically demanding were main reasons for not participating in outdoor activities, while an actual change of interest or lack of motivation was not mentioned to the same extent among our interviewees. Thus, we believe, in line with a person-centered approach, that it is important to identify the outdoor activities and which context the person actually wants to participate in. This to support older adults’ self-perception of his/her ability in outdoor recreation [59].

### Contextual factors

In line with the PEO model [44], it is essential that the intervention addresses the older adults’ level of functioning and how self-perception can be optimized. Simultaneously, the intervention should address how contextual factors in the environment can support engagement in outdoor activities.

Overall, there was a clear pattern that outdoor activities nearby home was important for most respondents. Environmental features in the neighborhood may provide support or hindrances for engagement in outdoor recreation [60]. Thus, the geographical location of residence is an important factor to consider since this may optimize the long-term opportunities for continued engagement in outdoor recreation. The active choice to move from a neighborhood with hills to a flat area, as reported by two interviewees, may have a significant impact on the opportunities for regular walks. This can be seen in relation to the proactive approach “anticipatory moves” proposed in the PCP model [43], i.e., to act and move to a less demanding dwelling with better accessibility. In a Swedish context, *residential reasoning*, whether to stay in a present dwelling or to move - has been described as an ambivalent process extended over time [61]. Therefore, a reflective component related to the location of residence should be included in an intervention aiming to promote outdoor recreation. Beyond the actual question of where to live, knowledge of the outdoor recreation opportunities could be considered e.g., in terms of where to find accessible areas for walking and the location of benches.

In our study, the engagement in outdoor activities with others was described in terms of support and to experience social participation. Different changes of the social context were events that had an impact on the opportunities for and meanings of outdoor activities. Examples of such changes were if a spouse or a friend had died or had begun experiencing functional limitations. Health changes among the people surrounding older adults, such as spouses and friends, are part of this social context. Thus, the social facet of outdoor engagement may be critical and need to be considered from the perspective of the unique person. If a social context has been important for someone’s engagement in outdoor recreation, the intervention needs to include how to re-establish a social context or how to adapt to a new social situation. It is hence relevant to discuss the importance of other social contacts and networks that could function as buffers to manage a period of loss and grief while maintaining outdoor recreation, as well as to provide motivation and practical support as to regain engagement over time. The local pensioners’ associations play an important role in terms of providing peer support and organized activities.

### Promoting subjective mobility needs

In our results, transportation did not seem to be a factor that had an impact on disengagement from outdoor recreation. One possible explanation might be that many of the respondents had access to the transportation needed to engage in outdoor activities. Also, most respondents considered nature areas nearby home as the most frequent context for outdoor recreation, thus reducing the need for transportation. However, while the home also may be a starting point for activities beyond the neighborhood, the potential means of reaching these activities needs to be considered. In a recent review [62], approximately one-third of older adults had unmet mobility needs. The pursuit of leisure, visiting friends and family was most associated with unmet travel needs [62]. Thus, there is a risk that over time, restricted mobility opportunities may negatively impact out-of-home outdoor activities. While out-of-home mobility is of great importance for health and well-being, there is a discrepancy between potential and realized mobility, i.e., the modes and mobility options a person has and the modes and mobility options modes she/he actually uses [63]. A recent study exploring the links between age and activity spaces explain how older adults have a tendency towards being active in more limited activity spaces compared to younger people. This can also be described in terms of life-space mobility (LSM) i.e., the spatial area in which a person moves in daily life, considering distance, frequency and assistance needed. Changes in LSM is of particular importance since a reduced life space is associated with poorer health outcomes and a decline in quality of life [64]. Thus, subjective mobility needs should not be neglected.

### Limitations

Since recruitment for the survey study was conducted during different events, we have no information about the representativeness of the study population. The empirical data collection was limited to one geographical location. Taken together, these limitations impact the generalizability of our findings. Part of the data collection was conducted during a period when restrictions due to the COVID-19 pandemic were present. Even though the restrictions in Sweden never were in the form of a lock down, people in general adhered to advice on physical distancing from the National Public Health Agency. This extreme situation might have affected the data collected and thereby the results and conclusions.

## Conclusions

By engaging in outdoor recreation, older adults have the potential to meet the needs related to meaning, purpose and social connectedness [31, 32] which therefore can affect health and well-being [34]. Our results verify that older adults are at risk for disengagement from nature and outdoor recreation and the associated risk of losing the health benefits associated with an active lifestyle. This process is described in terms of loss as and with a sense of acceptance. We propose a set of core features to be considered in the design of an intervention aiming to promote engagement in outdoor recreation for older adults and to reduce the risk for involuntary disengagement. These features are person-centeredness, promoting functioning, addressing self-ageism, providing environmental support, promoting subjective mobility needs and adaptation to find new ways to engage in outdoor recreation.

## Data Availability

The datasets analyzed during the current study are available from the corresponding author on reasonable request.

## Abbreviations

LSM: Life Space Mobility
PCP: Comprehensive Preventive Corrective Proactive model
SOC: Selection, Optimization, Compensation model
WHO: World Health Organization
